# Supplementary material for: Are carbon emissions trading and green financial instruments synergistic? -Comprehensive quantitative research based on content analysis
Source: PLoS One. 2024 Mar 7;19(3):e0298601. doi: 10.1371/journal.pone.0298601 (PMC10919601; doi:10.1371/journal.pone.0298601)
Supplement: S1 Data — (ZIP) [file pone.0298601.s001.zip › Data/地级市Z_F工作报告.zip!/║╙─╧/╨┼╤⌠╩╨/└┤╫╘╣1⁄2╓┌║┼í╛┬φ┐╦╩2╛▌═°í┐_2016.docx]

各位代表：

　　现在，我代表市人民政府，向大会报告工作，请予审议，并请各位政协委员和列席会议的同志提出意见。

　　一、“十二五”主要成就及“十三五”主要目标

　　“十二五”是我市经济社会发展极不平凡的时期。在省委、省政府和市委的正确领导下，全市上下全面落实党的十八大和十八届三中、四中、五中全会精神，深入贯彻习近平总书记系列重要讲话精神，扎实开展党的群众路线教育实践活动，主动适应经济发展新常态，充分发挥前沿、生态、民智三大优势，在抢抓机遇中持续求进，在爬坡过坎中务实发展，在蓄势崛起中积极作为，干成了一批打基础利长远的大事，取得了一些具有标志性意义的重大突破，胜利完成“十二五”规划目标任务。

　　综合实力迈上新台阶。2015年全市生产总值1878亿元，是2010年的1.6倍，总量居全省第9位，比2010年上升两个位次;一般公共预算收入91亿元、支出374亿元，分别是2010年的2.7倍和2.2倍;固定资产投资2024亿元，是2010年的2.6倍，投资规模居全省第4位;社会消费品零售总额880亿元，较2010年翻一番，总量居全省第5位;全市金融机构存款余额2375.4亿元、贷款余额1290.9亿元，均是2010年的2.3倍，总量分别上升到全省第4位和第6位。

　　转型发展取得新成效。突出抓好“一个载体、四个体系”建设，电子信息、绿色食品、新型建材、纺织服装等主导产业初具规模，现代家居、现代物流等产业集群加速集聚。交通银行、民生银行等多家金融机构入驻信阳，村镇银行实现县区全覆盖，多元化的金融体系初步形成。大力实施商标战略、名牌战略和质量立市战略，全市获得中国驰名商标9个、中国地理标志证明商标4个、国家地理标志保护产品3个、省长质量奖3个。科技创新能力显著增强，新增国家级高新技术企业18家，创建院士工作站7家，组建国家级产业技术创新战略联盟1家、省级4家。

　　城乡面貌呈现新气象。坚持以城带乡、城乡一体、协调并进，中心城市辐射带动能力显著增强，县城和中心镇加快发展，新型城镇化步伐持续加快，全市常住人口城镇化率达到42.7%，比“十一五”末提高8.3个百分点。成功创建国家级生态示范市、中国优秀旅游城市、国家园林城市、国家卫生城市、省文明城市和一批美丽乡村，连续七届蝉联中国十佳宜居城市，连续5届荣获中国最具幸福感城市。

　　厚植优势实现新提升。积极谋划重大专题，信阳被确定为全国农村改革试验区和国家首批主体功能区建设试点示范市;大别山革命老区振兴发展规划获得国务院批复，信阳被确定为三个核心发展区域之一;淮河生态经济带发展规划上升到国家战略研究层面，信阳作为淮河生态经济带的“三核”之一得到重点关注;信阳城乡一体化示范区获得省委、省政府批准。持续加强基础支撑体系建设，京广高铁贯通信阳，境内宁西铁路复线电气化改造、京港澳高速公路和312、107国道改扩建工程基本完成，息淮固高速公路建成通车，明港机场开工建设，淮河航运淮滨港吞吐能力显著提升，信阳海关建成通关，500千伏春申输变电工程建成投运，出山店水库开工建设。持续提升生态优势，全市森林覆盖率达到36.11%，大气和水环境质量位居全省前列。

　　改革开放赢得新局面。行政体制改革有序实施，非行政许可审批全面取消，商事登记制度改革全面推开，农村改革试验深入推进，财税金融、交通运输、社会事业等领域改革取得显著成效。成功举办“中国光彩事业信阳行”和第十届豫商大会等重大活动，促成了一批战略合作项目。“十二五”期间累计进出口32.7亿美元，是“十一五”的3.6倍;外派劳务3.5万人次，利用外资扩展到15个国家和地区，与124个国家和地区建立了经贸关系，26家国内外500强企业投资信阳。

　　社会事业步入新阶段。五年全市财政民生支出1146.5亿元，占财政总支出的75.7%。城乡居民人均可支配收入达到22434元和9844元，分别是2010年1.68倍和1.85倍。城镇累计新增就业49.5万人，农村劳动力转移就业新增45.8万人。城乡居民基本养老保险和大病保险制度实现全覆盖，社会救助制度全面建立，城镇企业退休人员基本养老金逐年提高，城乡低保标准比2010年提高81.8%和155.6%，新农合和城镇居民医保财政补助标准增长216.7%，农村五保对象集中供养、分散供养标准分别提高90%和133.3%。新建保障性住房9.06万套。教育事业得到优先发展，信阳农林学院成功升本，信阳学院获得批准。全市学前三年毛入学率达到82%、提高10个百分点，普通高考录取率达到97.7%、提高15.8个百分点。覆盖城乡的公共卫生和医疗服务体系基本建立，新增医疗卫生机构床位2873张。公共文化服务水平持续提升。全民健身运动广泛开展。新建改建农村公路3530.7公里，改造农村危房12.99万户，202.7万农村居民和57.5万农村学校师生饮水安全问题得到解决，64.3万贫困人口实现稳定脱贫。

　　各位代表!经过五年努力，信阳经济社会发展站在了新的起点，为全面建成小康社会打下了坚实基础。面对复杂的经济形势，我们取得的成绩来之不易。这是落实党中央、国务院和省委、省政府决策部署的结果，是市委坚强领导的结果，是市人大、市政协大力支持的结果，是全市人民团结拼搏的结果。在此，我代表市人民政府，向全市广大工人、农民、知识分子、干部职工，向解放军指战员、武警官兵、政法干警，向中直、省直机关驻信单位，向全市新闻工作者，向在我市创业的境内外投资者、建设者及社会各界人士，致以崇高敬意和衷心感谢!

　　经过“十二五”时期的探索实践，我们也积累了宝贵经验。主要是：始终坚持遵循规律、持续求进，紧紧扭住发展第一要务，主动融入全国全省发展大局，用正确的方法做正确的事情，牢牢把握发展主动权;始终坚持问题导向、务实发展，把工业化、城镇化作为现阶段的突出任务，统筹促增长、调结构、转方式，加快推进“四化”同步科学发展;始终坚持责任担当、积极作为，着力打基础利长远、兴产业带全局、强实力惠民生，让人民群众共享改革发展稳定成果。这些经验是全市人民智慧的结晶，弥足珍贵，应当倍加珍惜、发扬光大。

　　各位代表!五年的成就和经验，充分彰显了全市各级组织的战斗力和创造力，提高了广大干群的凝聚力和向心力，增强了信阳人民的自信心和自豪感，必将激励我们乘势而上、持续前行!

　　“十三五”是我市发展机遇集中体现的时期，是各种优势潜力有效释放的时期，是破瓶颈补短板的紧要时期，是持续加快发展、力争弯道超车的关键时期。机遇承载使命，挑战考验担当，我们必须在新的起点上奋力开创新局面。

　　根据市委建议，市政府编制了《信阳市国民经济和社会发展第十三个五年规划纲要(草案)》，已印发会议，提请审议。

　　“十三五”时期经济社会发展的指导思想是：高举中国特色社会主义伟大旗帜，全面贯彻落实党的十八大和十八届三中、四中、五中全会精神，以马列主义、毛泽东思想、邓小平理论、“三个代表”重要思想、科学发展观为指导，深入贯彻习近平总书记系列重要讲话精神，按照“五位一体”总体布局和“四个全面”战略布局，坚持发展为第一要务，牢固树立和贯彻落实创新、协调、绿色、开放、共享的发展理念，以提高发展质量和效益为中心，加快形成引领经济发展新常态的体制机制和发展方式，推进“四化”同步科学发展，强化“一个载体、四个体系、六大基础”战略支撑，认真落实河南省三大国家战略规划和大别山革命老区振兴发展规划，大力承接产业转移，着力构建区域性中心城市，充满发展活力的生态经济先行区，全国有影响力的交通、信息、物流枢纽，着力保障和改善民生，持续求进，务实发展，积极作为，确保如期全面建成小康社会，为全面实现现代化奠定坚实基础。

　　主要预期目标是：经济保持较高速度增长，生产总值年均增速高于全省平均水平1个百分点以上，经济社会发展主要人均指标达到或接近全国平均水平，转型升级和创新驱动实现新突破，人民生活水平和质量普遍提高，全民素质和社会文明程度显著提高，生态优势持续提升，治理体系和治理能力现代化迈出坚实步伐，到2020年全面建成小康社会。

　　实现上述目标，必须积极推动创新发展，全面促进协调发展，持续提升绿色发展，大力实施开放发展，更好实现共享发展。

　　各位代表!“十三五”发展蓝图已经绘就，决胜全面建成小康社会的号角已经吹响。我们坚信，在省委、省政府和市委的正确领导下，在全市人民的团结拼搏中，“十三五”确定的目标一定能够实现!信阳人民的生活一定会更加美好!

　　二、2015年工作回顾

　　2015年是“十二五”的收官之年。我们按照省委、省政府和市委的决策部署，深入开展“三严三实”专题教育和“三查三保”活动，统筹抓好稳增长、促改革、调结构、强基础、惠民生、防风险各项工作，一以贯之地持续求进、务实发展、积极作为，较好完成了市四届人大五次会议确定的目标任务，主要经济指标增速位居全省前列。全市生产总值增长8.9%，增速高于全省0.6个百分点;一般公共预算收入增长13.3%，增速居全省第1位;规模以上工业增加值增长10.1%，增速居全省第4位;固定资产投资增长17.5%，增速居全省第6位;社会消费品零售总额增长13.1%，增速居全省第3位;出口总值增长15.3%;城乡居民人均可支配收入分别增长9.1%和9.9%。一年来，我们主要做了以下工作：

　　(一)紧紧围绕促增长保态势，持续加快经济发展。坚持把促增长保态势作为全局工作的突出任务，认真落实中央和省市各项促增长政策措施，大力开展“解难题促增长”百日攻坚行动和“争先进比贡献”竞赛活动，全市经济运行稳中有进、稳中提质。着力扩大有效投资。坚持重大项目联合督察，全市97个省市重点项目完成投资266.2亿元，占年度目标的111.8%。坚持产业集聚区重点项目攻坚督导协调例会制度，集中解决产业集聚区发展中的困难和问题，114个重点攻坚项目完成投资160.4亿元，占年度目标的107.9%。着力增加社会需求。积极发展消费信贷，培育消费热点，着力增强消费对经济增长的拉动作用。坚持以鼓励住房消费与推进城镇化、消化商品房库存与增加保障性住房供给相结合，持续推进房地产业健康发展。房地产投资、销售分别增长17.7%和11.1%。着力服务企业发展。突出重点行业、重点企业，深入开展产销对接、用工对接、银企对接、产学研对接，为企业解决融资76.3亿元、用工1.6万人，帮助企业降本增效、延长链条、开拓市场、转型解困。全市规模以上工业企业利润增长20%，高于全省20.1个百分点。着力强化要素保障。设立信贷风险补偿资金，引导金融机构支持实体经济发展。全市金融机构存贷款余额分别增长13.4%和16.5%，增速分别居全省第4位和第5位，存贷比达到54.3%、同比提高1.4个百分点，新增贷款183亿元。资本市场融资68.6亿元。科学使用计划指标，充分运用城乡增减挂钩政策，全年争取省政府批准建设用地3万多亩。持续加强电网建设，完成新一轮农村电网改造升级工程，产业集聚区实现110千伏及以上变电站全覆盖。

　　(二)紧紧围绕调结构转方式，持续推进转型升级。大力发展现代工业。更加“关注工业、学习工业、研究工业、支持工业”，全市170个重大产业结构调整项目完成投资250.4亿元，占年计划的105.6%，已建成投产项目51个;规模以上工业企业主营业务收入、利税分别增长10.4%和17.5%;高成长性制造业和高技术产业增加值分别占规模以上工业企业的55%和10%，同比提高3.3个和1.6个百分点。大力发展现代服务业。金融服务、信息服务、文化旅游等高成长服务业和健康养老、科技服务、电子商务、物流快递等新兴服务业快速发展。全年接待游客2525万人次，实现旅游收入145.2亿元，分别增长13.6%和14.5%，我市创建国家5A级旅游景区和国家生态旅游示范区工作扎实推进;电商物流业蓬勃发展，全市规模以上快递服务企业业务量累计完成1277.4万件，增长74.9%，电子商务交易额突破70亿元，“双十一”期间日均快递业务量居全省第2位。信阳成为2016年全省除郑州外唯一增加邮路的城市，光山县被确定为全国电子商务进农村综合示范县。大力发展现代农业。深入实施“三农”三大工程，持续推进农业综合开发，新建高标准粮田65万亩，主要粮食耕种收综合机械化水平达到68%，全年粮食产量119.4亿斤、再创新高。畜牧业和水产业健康发展，肉蛋奶和水产品产量稳定增长。积极培育新型农业经营主体，推进农业适度规模经营，建成省级农业标准化示范区19个，市级以上农业产业化集群25个、农业产业化重点龙头企业345家。农村土地承包经营权确权登记颁证工作基本完成。

　　(三)紧紧围绕打基础强支撑，持续增强发展后劲。加快推进发展载体建设。大力实施产业集聚区“四大工程、三大计划”，全市有6个产业集聚区晋升为一星级产业集聚区，信阳市产业集聚区和潢川经济技术产业集聚区分别被授予全省十先进、十快产业集聚区。信阳电子信息产业集聚示范区被命名为中国手机生产基地，已集聚规模以上电子信息企业89家;国际家居产业小镇被命名为中国(信阳)新兴家居产业基地，已签约项目63家，开工建设30家，建成投产10家;金牛物流产业集聚区荣获中国电子商务物流服务模式创新奖;上天梯被确定为国家绿色建材高新技术产业化基地。全市产业集聚区完成固定资产投资980亿元，增长15.3%，占全市的55.6%;规模以上工业企业主营业务收入突破1700亿元，增长12.7%，占全市的82.8%，产业集聚区的综合带动作用日益增强。加快构建现代产业体系。突出围绕主导产业，集中一切要素资源，引龙头、抓延链、建基地、育集群，不断壮大高成长性产业、改造提升传统优势产业、培育发展战略新兴产业。我市电子信息、新型建材、绿色食品、现代家居、现代物流等主导产业总规模突破1200亿元。加快构建现代城乡体系。组织编制信阳市城市总体规划(2015-2030年)，统筹推进中心城市、县城、中心镇和新农村协调发展。大力实施新型城镇化三年行动计划，强化“一基本两牵动三保障”，有序促进了一批农村劳动力进城转移。加强城市精细化、数字化、市场化管理，城镇化水平和质量持续提高。加快构建自主创新体系。实施了一批重大专项，新增国家高新技术企业8家，新增院士工作站1家，创建省级重点实验室2家。在首届中国“互联网+”大学生创新创业大赛总决赛中，我市三个团队获得银奖，占全省三分之一。加快构建现代市场体系。落实工商登记便利化，全市新登记市场主体3.19万户，增长23.76 %。积极培育新业态新模式，全市商务中心区和特色商业区完成投资69亿元，鄂豫皖一日达物流产业园等重大项目加快建设。深入推进万村千乡市场工程建设，新建一批社区平价超市。充分发挥 “三市一库”和实训基地就业保障作用，规范公共资源交易中心建设，进一步完善要素市场，促进了创业就业。

　　(四)紧紧围绕保生态优环境，持续提升发展优势。坚持把生态优势作为信阳可持续发展的最大竞争力，将生态文明理念融入城市建设和经济社会发展全过程。加强环境保护。开展国家主体功能区试点示范市建设，持续实施大别山生态安全屏障、淮河上游水生态建设和林业生态市建设提升工程，加强淮河源生物多样性保护与可持续利用，推进生态县创建工作，成功通过国家园林城市复检和全国绿化模范城市验收。全市完成造林27.5万亩、森林抚育和改造46.1万亩。推进综合治理。以治理灰霾为重点，深入实施蓝天工程、碧水工程、乡村清洁工程和城市河流清洁行动计划。加强重点区域和行业环境综合治理，全面推行农作物秸秆禁烧，淘汰黄标车16952辆，完成99台燃煤锅炉和346座加油站拆改任务，空气质量位居全省前列;加强饮用水源地保护，落实最严格水资源管理制度，城市饮用水源地水质达标率100%。持续加强农村面源污染和土壤污染治理，综合整治农村环境，平桥区郝堂村荣获中国人居环境范例奖。强化节能减排。认真落实环境准入制度，优化环境资源配置，实施一批循环经济、清洁生产和节能节水示范工程，推进建筑节能及城市“禁黏”和乡镇“禁实”，促进资源节约集约利用。省下达的节能减排目标全面完成。

　　(五)紧紧围绕抓改革促开放，持续培育发展动力。深化重点领域改革。积极跟进落实中央和省市决策部署，一些重要领域和关键环节改革取得重大进展。着力转变政府职能，市政府工作部门权责清单全部建立，清理规范行政审批中介服务事项全面展开，“三证合一、一照一码”改革全面落实，事业单位分类改革前期工作初步完成。户籍制度、财税金融和投融资体制改革有序实施，农村改革试验特别是四项试点改革向纵深推进，林权、水利、供销、农电和社会事业等体制改革持续深化，党政机关公务用车制度改革稳步推进，不动产统一登记制度改革全面启动。明港镇被确定为国家建制镇示范试点。大力推进开放招商。积极推进与三大电信运营商、铁塔公司的深度合作，全面加强与金融机构的战略合作，持续深化与战略投资者的互惠合作，全年实际利用外商直接投资4.9亿美元、实际引进省外投资194.7亿元，分别增长11%和9 %。成功举办信阳第23届国际茶文化节、第十一届中国茶业经济年会、中国二维码产业促进工程大会等，信阳对外开放水平不断提升。

　　(六)紧紧围绕惠民生保稳定，持续促进社会和谐。扎实推进省十项重点民生工程和市“十件实事”建设，全市财政民生支出293.8亿元，增长16%。扎实推进精准扶贫。突出抓好扶贫对象的精准管理、扶贫资金的精准使用、贫困家庭的精准脱贫、特殊群体的精准帮扶和贫困村的精准扶持，全市又实现11万人稳定脱贫，出山店水库移民安置和淮干滩区居民迁建工作进展顺利。努力促进就业创业。实施积极的就业扶持政策，支持创业带动就业，全市城镇新增就业10万人，失业人员实现再就业3.53万人，就业困难人员实现就业1.39万人，均超额完成全年目标。持续加强社会保障。信阳被确定为全国电子社保示范城市。城镇基本医疗保险实现市内异地就医直接结算。企业退休人员养老金人均月增208元，城镇居民基础养老金月人均提高到78元，城乡低保对象年补助标准分别提高到不低于2700元和1380元，农村五保对象年集中供养、分散供养标准分别提高到不低于3800元和2800元。养老服务体系逐步建立，新增城乡社区养老服务中心69所，新增社会养老机构4家，新增床位656张。基本建成保障性住房及棚户区改造住房13696套，改造农村危房29600户。法律援助覆盖面扩大到全市40%以上人口，免费为困难群众办理案件4114件。大力发展社会事业。持续加强学前教育，改善农村贫困地区义务教育薄弱学校办学条件，实施扩充城镇义务教育资源五年计划，全市公办幼儿园和普惠性民办幼儿园覆盖率达到82%，义务教育均衡发展，高中教育和职业教育质量位居全省前列，空军招飞人数连续3年位居全国地级市第一。信阳通过国家二类城市语言文字评估验收并达到优秀等级，我市选手徐玮琦在全国汉字听写大会上代表河南省夺得全国总冠军。贫困家庭学生资助实现全覆盖。大力发展卫生计生事业，医疗卫生服务体系持续完善，开工建设一批新的医院。县级公立医院综合改革全面推开，药品加成全面取消。人均基本公共卫生经费提高到40元，新农合和城镇居民医保人均财政补助标准提高到380元。农村居民重大疾病医疗保障病种扩大到35种，实际报销比例达到75%。我市被确定为新农合跨省“即时结报”试点市。落实全民健身国家战略，成功举办第七届全国自行车公开赛等重大赛事。新建一批文化惠民工程。着力改善生产生活条件。实施城镇基础设施扩能增效工程，推进城乡基础设施一体化建设。中心城区新增100辆新能源公交车，新建和改造供水管网46公里。全市新增燃气用户29480户。投入运营污水处理厂9座、生活垃圾无害化处理场8座，新增污水日处理能力21.5万吨，日处理生活垃圾1870吨，县城垃圾无害化处理率达90%以上。4G网络实现县城和乡镇点全覆盖，宽带光纤到户覆盖22万户。扎实推进美丽乡村建设和农村人居环境改善工作，创建896个达标村和412个示范村，解决低电压用户24万户，改建农村公路1144.8公里，新建、改造桥梁2642.1延米，完成270座病险水库除险加固、11条中小河流治理和3个大中型灌区续建，解决57.8万农村居民和18.5万农村学校师生饮水安全问题。积极创新社会治理。持续深化法治信阳、平安信阳建设，加强社会治安综合治理，依法打击违法犯罪活动;推进信访工作制度改革，发挥民声手机短信平台作用，及时化解社会矛盾，依法解决信访群众诉求;严格落实安全生产责任，全面排查整治安全事故隐患，深入开展食品药品和重点消费品质量安全专项治理，依法打击处置非法集资，不断完善公共安全应急管理体系，全市安全生产形势总体平稳，群众安全感、满意度持续上升。

　　一年来，我们自觉接受人大监督、政协民主监督和社会监督，认真办理人大代表建议和政协提案，支持民主党派、工商联和无党派人士参政议政。全面加强依法行政和法治宣传教育，深入推进服务型执法，落实行政执法责任制，深化政务公开，法治政府建设取得新进展。大力开展精神文明创建活动，先后有6人获得全国道德模范及提名奖，数量位居全国地级市前列;2015年有1人被评上全国十佳农民，1人被评为全国好警嫂，2人被授予河南省感动中原十大人物，“信阳好人”现象引人注目。持续加强国防教育、国防后备力量及人民防空建设，“双拥”工作深入推进，军民融合深度发展，海军信阳舰入列命名，涉军维权的“信阳模式”被最高人民法院总结推广，驻信解放军、武警部队和民兵预备役人员为地方发展作出新贡献。工会、共青团、妇联和科协、文联、社科联、残联、红十字会等人民团体在经济社会发展中发挥了重要作用，民族、宗教、外事、侨务、对台工作全面加强，统计、物价、机关事务、地质、气象、防震减灾、人防、档案、史志等工作取得新成绩。

　　各位代表，我们也清醒地认识到，经济社会发展中还存在一些突出问题。经济运行中的不确定、不稳定因素依然较多，部分行业和企业生产经营还十分困难，国企改革进展缓慢、任务艰巨;工业化城镇化水平不高，城乡之间、县区之间发展还不均衡，三次产业、三大需求之间结构还不协调，创新能力不强，开放的广度深度不够;脱贫攻坚任务较重，公共服务欠账较多，教育、医疗、养老、住房、环境治理等与人民期待还有较大差距;政府职能转变还不到位，部分公务人员的履职担当能力和服务效能还比较差，法治政府建设和反腐倡廉工作有待进一步加强。对此，我们一定高度重视，主动担当，认真解决，努力让人民满意!

　　三、2016年重点工作

　　今年是实施“十三五”规划的开局之年，做好政府工作十分重要。当前，世界经济在深度调整中曲折复苏，我国经济长期向好的基本面没有改变，国家着力把中原地区培育成为带动发展的新空间，河南在全国发展大局中的战略地位更加突出。经过多年发展，我市政策环境、基础支撑和综合优势总体好于“十二五”时期，加之去产能、去库存包袱小，新产业新业态新模式带来的发展潜力大，完全有条件实现更大发展，打赢供给侧结构性改革攻坚战。

　　今年经济社会发展的主要预期目标是：生产总值增长9%左右，一般公共预算收入增长9%，全社会固定资产投资增长12%，社会消费品零售总额增长12%，进出口总值增长7.5%，实际到位省外资金增长7.5%，城镇新增就业9万人以上，城乡居民收入增长与经济增长基本同步，居民消费价格涨幅3%左右，节能减排和环境质量改善等约束性指标完成省下达任务，发展质量和效益明显提高，结构性改革取得实质性进展。

　　生产总值预期增长9%左右，充分考虑了我市发展需要和客观条件，权衡了各种因素。从必要性上看，我市人均生产总值不到全省平均水平的八成，只有增速快于全省平均水平，才能逐步缩小差距，与全省同步全面建成小康社会。从可行性上看，我市发展基础不断夯实，支撑条件不断提升，积极因素持续放大，增长动能不断释放，增长趋势和市场预期持续向好，只要我们坚定不移地按照省委省政府和市委的决策部署，坚持加快发展不动摇、坚持脱贫攻坚不动摇、坚持生态保护不动摇、坚持改革开放不动摇、坚持改善民生不动摇，同心同德，务实重干，完全可以实现这一目标，确保“十三五”开局得胜。

　　今年重点做好八个方面工作：

　　(一)突出结构性改革，着力提高发展质量效益。立足我市所处阶段的历史任务和主要矛盾，着力优化投资结构、产业结构、供需结构，增强经济发展内生动力。

　　持续扩大有效供给。围绕省重点支持的十大产业，选准我市主攻方向，精准发力，积极作为。聚焦主导产业，持续做大增量。突出电子信息、绿色食品、新型建材、现代家居、现代物流、纺织服装等主导产业，大力实施优势产业链培育工程，培育壮大新的经济增长点。坚持以龙头带动、延链补链、集群配套为抓手，拓宽延长智能终端产业链条，加快做强电子信息产业;以承接家具、厨卫等产业链式转移、集群发展为重点，加快做大现代家居产业;以主食产业化和粮油精深加工、畜禽深加工为重点提升绿色食品优势产能;以技术突破、应用推广为重点发展壮大节能环保产业;以服装、鞋帽、羽绒和家用纺织品加工为重点，推动纺织服装产业升级;以提升研发能力、扩大市场规模为重点，推动医药制造产业加速发展;以引进高新技术和先进设备为重点，改造提升传统制造业。大力发展现代服务业，持续提升水平。围绕扩大总量、提高效率、增强辐射，大力推进金牛物流产业集聚区等物流园区智能化、信息化、标准化建设，加快现代物流体系建设，加速人流、物流、资金流、信息流集聚，努力把信阳建成全国重要的区域性电商物流组织中心。大力发展现代金融业，积极构建普惠金融体系，不断改进对薄弱领域、弱势群体的金融服务，增强金融服务便捷性。突出培育主体、集聚带动，推动发展互联网金融、绿色金融、金融租赁等新兴业态，逐步提高金融业增加值占服务业比重，着力打造区域性金融服务中心，带动生产性服务业向专业化转变、向价值链高端延伸。加快发展生活性服务业，积极实施养老健康产业推进计划，推动生活性服务业向精细化、高品质提升。持续提升旅游业，加强旅游基础设施建设，推进国家5A级旅游景区和国家生态旅游示范区创建工作，大力发展乡村旅游和特色旅游，打造国际山地休闲旅游度假区、茶文化体验区、大别山红色生态旅游区等旅游目的地。提升体育产业，发展健身休闲、户外运动、竞赛表演等体育服务业，打造一批全国性、区域性体育赛事。提升特色餐饮业，着力打造信阳茶、信阳菜等特色品牌，扩大行业影响力。提升房地产业，继续把鼓励住房消费、加快消化库存作为促进房地产业健康发展的首要任务，以满足新市民住房需求为主要出发点推进住房制度改革，推进房地产业健康有序发展。降低企业成本，持续优化存量。全面落实国家降低企业税费负担、社会保险费、电力价格等政策，充分运用企业金融债务重组相关规则，实施涉企经营服务性收费目录清单管理，降低企业生产经营和融资成本。鼓励企业开展上下游兼并重组，提高产业准入能耗、物耗、水耗和生态环保标准，促进企业产品升级，加快向高端、智能、绿色方向转型转产。继续开拓产能利用空间，扩大产销对接，支持企业利用“互联网+”等方式，拓展国内外市场。积极争取上级政策，加强分类指导，推动产能过剩行业重组，促进企业转型解困，有效处置特困企业。

　　持续挖掘需求潜力。发挥投资对增长的关键作用。积极对接我省 “1816”投资促进计划，争取我市有更多项目进入计划篮子，抓紧启动一批条件成熟的重大工程;加快实施160个重大产业结构升级项目和169个服务业重点项目，力争全年完成投资440亿元。统筹做好专项建设基金项目申报和实施工作，确保去年争取的24个项目全部开工建设。持续加大重大项目谋划，加快编制三年滚动投资计划，力争储备项目达到1000个，总投资超过400亿元。大力争取中央预算内资金和省级资金，积极推广PPP模式。发挥消费对增长的基础作用。启动新兴热点消费促进工程，持续扩大旅游休闲、教育培训、健康养老、家政服务、文化体育等服务消费能力，大力推进跨区跨境、线上线下、体验分享、电子商务、智能家居等信息消费，积极培育绿色消费，持续提升农村消费。进一步完善提升社区平价超市。发挥进出口对增长的促进作用。进一步优化大通关机制，积极引进外向型项目，培育做大电子、茶叶、水产、禽肉等出口基地和外向型产业集群，支持优势企业走出国门。支持先进技术设备、关键零部件和其它需求旺盛的产品进口。

　　(二)突出创新发展，着力增强经济发展动力。加快提升发展载体。将产业集聚区作为推进结构性改革的示范区，持续推进产业集聚区提质创新发展，积极承接和大力发展高端装备制造业、战略新兴产业、中高端消费品工业。持续推进产业集聚区“争星晋位”计划、重点项目攻坚计划、优势产业集群培育工程、“四一两化”招商行动，不断推动产业集聚区上规模上水平上层次，提高吸引力竞争力带动力。进一步推动商务中心区提升生产性服务功能、特色商业区提升生活性服务功能，积极支持信阳高新区和潢川经济技术开发区创建国家级高新区和经济技术开发区。产业集聚区固定资产投资和规模以上工业企业主营业务收入增速要高于全市，力争有10个以上产业集聚区达到一星级标准。加强科技创新支撑。支持企业加强与央企、科研院所、高等院校深度合作，促进产业技术创新战略联盟协同创新，实施一批省级重大科技专项，突破一批制约我市主导产业链式发展的关键核心技术。全面推进质量立市战略，支持企业创建国家级技术研究中心、工程(技术)研究中心和工程重点实验室，加快国家岩石粘土类非金属矿产品质检中心及省级木质家具质检中心、省级羽绒制品检验检测中心建设，积极推进国家级食品检验检测平台建设，推动产业不断创新升级。推动大众创业万众创新。发挥“互联网+双创”的乘数效应，积极发展众创、众包、众扶、众筹，促进生产与需求对接、传统产业与新兴产业融合，形成线上与线下协调的创业创新格局。充分发挥各种研发平台、创业创新服务中心和创业创新实践基地的作用，搭建开放共享的创业创新基础性平台，形成更多开放便捷的众创空间。鼓励引导科技人员、高校毕业生、返乡农民工、退役军人等重点人群创业创新，积极营造支持鼓励创业创新的社会氛围。大力发展互联网经济。实施“互联网+”行动计划，促进信息化与新型工业化融合创新，加快工业互联网应用，推进企业装备智能化、生产自动化、商务电子化，推动基于互联网的产品设计、柔性制造、个性化定制等新型模式，培育战略新兴产业。推动互联网与现代农业跨界融合，加快物联网技术在农业领域的应用，推进农业生产精确化、高效化。促进互联网与现代服务业深度融合，推动交通运输、商贸物流、餐饮家政、育幼养老、在线教育、金融等公共服务领域资源整合和优化配置，促进民生和社会领域信息化应用，催生新的消费增长点。积极引进互联网行业龙头企业设立分支机构，支持信阳互联网企业加快发展。

　　(三)突出城乡统筹，着力推进新型城镇化。发挥新型城镇化“一发动全身”的带动作用，坚持“一基本两牵动三保障”，促进城乡发展一体、区域发展协同。加快构建现代城镇体系。加强与周边城市协作，全面推进交通一体、产业链接、服务共享、生态共建、发展共赢，加快建设区域性中心城市。以信阳市城市总体规划(2015-2030年)为指导，全面提升城市规划建设管理水平，统筹推进中心城市、县城和中心镇协调发展，加快推进新区开发、老城改造、产业集聚区建设和“6+3”中心城市组团发展，启动信阳城乡一体化示范区规划建设，支持明港镇加快发展成为具有一定规模的城市。加快编制县域城乡总体规划、村庄规划和城市近期建设规划，推进多规合一。全面开展城市设计，加强对建筑设计、城市风貌的约束和指导，着力构建山水相间、疏密有致、开合有度、生态宜居、具有文化内涵与品质的空间格局和城市风貌。加快推进农业转移人口市民化。出台我市推进三个一批人城镇化实施方案，加快户籍制度改革和居住证制度“双落地”，研究出台促进农民举家进城落户的政策措施，统筹解决就业、就医、就学、住房等问题，维护进城落户农民土地承包权、宅基地使用权、集体收益分配权。提高城镇综合承载能力。统筹加快城市基础设施建设，系统解决城市发展中的关键问题，持续提高城镇综合承载能力和匹配度。加快新老城区的道路衔接，再打通一批断头路，改造提升一批主次干道和背街巷道。优先发展城市公共交通，加强停车场站和公共自行车等设施建设，着力缓解城市交通拥挤压力。继续实施一批城市内河治理、污水处理、垃圾处理、供排水、防洪、燃气等基础设施建设，加快推进地下空间综合利用和综合管廊建设。严格执行城市蓝线管理办法，建设海绵城市和生态低碳城市。完善城市管理综合执法体制机制，推动执法重心下移和属地化管理。深入开展全国文明城市创建工作，持续提升创建成果，完善长效机制，全面提高市民综合素质和城市文明程度。

　　(四)突出固本强基，着力发展现代农业。坚持集约高效绿色可持续发展，促进农业增效、农民增收、农村繁荣。提高粮食生产能力。认真落实粮食安全责任制，落实藏粮于地、藏粮于技战略，提升农业机械化水平，靠科技创新引领农业发展。划定永久基本农田并实行特殊保护，实施耕地质量保护与提升行动，统筹推进土地整理、中低产田改造、新增粮食生产能力规划田间工程、小农水重点县等项目建设，新建高标准粮田58万亩，确保粮食总产稳定在现有水平以上。大力实施主食产业化和粮油深加工工程，促进我市由产粮大市向粮食经济强市跨越。转变农业发展方式。加快农业科技创新与成果转化，大力发展特色农业、高效农业、绿色农业和品牌农业，增加市场紧缺农产品生产，提高农产品供给质量、效益和安全水平。加快培育现代农业产业化集群，大力发展都市生态农业，实施种养结合循环农业示范工程，推动粮经饲统筹、农林牧渔结合、种养加一体、一二三产融合发展。深化农村改革试验。继续抓好涉农领域改革，扎实推进集体林权制度改革、改进农业补贴办法、涉农建设性资金整合和扶贫开发综合改革试点，引导金融资本和社会资本进入，激发农业农村发展活力。大力发展专业大户、家庭农场、农民专业合作社和农业产业化重点龙头企业等新型经营主体，培育新型职业农民，健全农业公共服务和社会化服务体系，鼓励发展多种形式的适度规模经营。改善农村生产生活条件。加快城镇基础设施和公共服务向农村延伸，完成农村公路三年行动计划乡村畅通工程，新建改建县乡公路300公里、村道500公里。持续开展美丽乡村建设，加强农村人居环境综合整治，推进农村生活垃圾治理五年专项行动，逐步实施改房、改水、改厕、改能、改路“五改”工程，改造农村危房1万户。

　　(五)突出厚植优势，着力强化基础支撑。持续加强基础支撑体系建设，提升发展保障能力。加强重大基础设施建设。积极推进京九客专、宁西客专信阳段等重大工程列入国家“十三五”规划，力争开工建设息县至邢集高速公路，积极对接淮滨至豫皖界高速公路，全力推进濮阳至湖北阳新高速公路信阳段和淮滨至息县航运工程等重大项目前期工作，加快建设明港机场，积极发展通用航空，实施一批道路、场站等基础设施项目，进一步提升信阳交通物流枢纽地位。加快推进出山店水库建设，力争开工建设淮干一般堤防加固工程，积极谋划张湾水库、引淮供水灌溉工程等大型水利项目。做好我市电网建设和新一轮农村电网改造升级工作，积极推进光山五岳抽水蓄能电站项目开工建设，加快建设西气东输二线南阳至信阳天然气管道工程，配合做好宁东—绍兴、昌吉—古泉特高压直流输电工程建设。加强与三大通信集团和铁塔公司的战略合作，推进电信基础设施共建共享，加快高速宽带网络建设，推动智慧城市、无线城市、光网城市建设，着力构建区域性信息枢纽。加强人力资源保障。以实现更高质量就业为目标，持续实施全民技能振兴工程，完善“三市一库”和实训基地建设，搭建劳动力就业服务新平台，推动技能培训再上新台阶。实施高层次科技人才、管理人才引进计划，强化企业家队伍建设，进一步提升人才支撑能力。继续创新人才培养选拔、评价使用、流动配置和激励保障机制，着力创造有利于吸引人才、培养人才、留住人才的环境，引导科技人才、工程技术人才向产业一线集聚。

　　(六)突出改革开放，着力提升发展活力。全面深化重点改革。深化行政体制改革，完成政府机构改革，加快事业单位改革，推进经济发达镇行政管理体制改革。加快国有企业改革，全面建立国有资产出资人监管权力清单和责任清单。深化商事制度改革，放宽市场准入，大力发展非公有制经济，保护各种所有制经济产权和合法权益。深化投融资体制改革，创新政府与社会资本合作项目收益和分配机制，开展有稳定收益的特许经营项目示范。探索设立产业发展扶持基金。深化财税金融体制改革，做好税收征管体制综合改革试点工作，推进营改增改革、消费税改革和资源税费改革，完善预算管理和非税收入管理改革、国库集中支付制度、政府采购管理和政府债务风险防控改革。强力推进农村信用社组建农商行等金融改革，大力吸引境内外银行、证券、保险机构在信阳设立分支机构，推动企业上市、挂牌、发行债权，支持上市公司再融资。深化文化体制改革，组建信阳日报传媒集团和广电传媒集团，推进传统媒体与新兴媒体融合发展，实现广播电视节目有线、无线数字化全覆盖。全面深化供销合作社综合改革。继续统筹抓好教育、科技、医疗卫生等领域改革。持续扩大开放招商。着力加强与“一带一路”沿线国家资源开发、农业、旅游等合作，支持有条件的企业开展国际产能和装备制造合作，参与沿线国家基础设施建设。持续打造开放平台，争取设立信阳铁路口岸、河南(信阳)水运口岸和信阳保税物流中心，推动海关跨区域合作、关检协作，建设内陆直通式海关。持续创新招商方式，积极参加我省产业转移专项对接等重大活动，举办好第24届信阳茶文化节。持之以恒推进“四一”招商，更加有针对性地开展集团化体系化招商，力争引进一批龙头型、基地型、集群型、支撑型重大项目，着力承接同一生态条件下的关联企业抱团转移、集群发展。持续强化项目落地，加大重点项目攻坚督导，进一步提高合同履约率、资金到位率和项目开工率。持续优化发展环境，从过去拼资源拼政策向比环境、比服务、比保障转变，坚持引进外地企业、支持入驻企业与培育壮大本土企业并重，确保项目落得下、建得好、发展快。

　　(七)突出环境保护，着力加强生态建设。牢固树立绿水青山就是金山银山的发展理念，突出发展、保护两手抓，继续实施蓝天工程、碧水工程、乡村清洁工程和城市河流清洁行动计划，不断改善和提升生态环境质量。强化综合治理。以治理灰霾和解决损害群众健康突出环境问题为重点，全面加强大气污染治理，突出抓好工业污染源防治、机动车污染控制、城市施工和道路扬尘治理，加大农作物秸秆禁烧及综合利用、燃煤锅炉拆改、油气回收治理和老旧车淘汰力度。加快城镇生活污水处理设施提标改造和配套管网建设，坚决消除黑臭水体。加大对河砂采集的整顿和规范管理。深入推进农业面源污染治理和土壤污染治理。严抓源头防控。完善污染企业退出机制。实施能源消费总量、强度双控制和节能环保等重大工程。推进建筑能效提升和绿色建筑全产业链发展计划。落实最严格水资源管理制度，加快实施一批循环经济、清洁生产和节能节水示范工程，推进资源节约集约利用。保护生态环境。持续实施林业生态市建设提升工程，推进退耕还林、天然林保护、湿地保护、重点地区防护林、生态廊道、生态修复等重大生态工程建设，加大废弃矿山植被恢复和保护，完成植树造林30万亩、森林抚育47万亩。完善长效机制。严格执行我市产业发展和区域开发环境保护负面清单，全面强化环境准入制度，进一步完善生态保护红线管理制度和污染防治区域联动机制，建立健全覆盖所有固定污染源的企业排放许可、网格化监管、评价考核与责任追究制度，精心呵护好信阳的青山绿水，坚决保护好我们的美丽家园!

　　(八)突出共享发展，着力增进人民福祉。加大基本民生支出，加强薄弱环节建设，提升基本公共服务，持续推进共享发展。打好脱贫攻坚战。全面落实大别山革命老区振兴发展规划，推进大别山区域一体化发展。坚持“五条途径”，落实“五项措施”，强化“五个保障”，健全脱贫攻坚联动机制，完善定点扶贫机制和选派第一书记驻村帮扶机制，支持银行业对接扶贫卡单、单列信贷资源、单独考核扶贫绩效，扎实有效地推进精准扶贫、精准脱贫，确保全年13.2万人实现稳定脱贫。稳定扩大就业。继续实施高校毕业生就业促进和创业引领计划，强化困难人员就业援助，支持农村劳动力转移就业和农民工返乡创业，确保实现城镇新增就业目标。多渠道增加农民经营性、工资性和财产性收入，依法治理拖欠农民工工资问题，坚决维护农民工合法权益!强化社会保障。全面实施全民参保登记计划，推动社会保障由制度全覆盖向法定人员全覆盖。加快电子社保示范城市建设和社会保障卡应用工作，全面推进“五险合一”。完善养老金合理调整机制，深化机关事业单位养老保险制度改革。统筹推进城乡社会救助体系建设，全面落实城乡低保、大病医疗救助、临时困难救助、农村“五保”供养等政策。做好养老事业和关爱“三留守”人员工作。扩大住房公积金制度覆盖范围。加大保障性安居工程建设，继续实施农村危房改造，切实解决低收入群体住房困难。抓好“菜篮子”工程，保障好人民生活。持续提高各项社会保障补助标准，坚决织密社会保障安全网!提高教育水平。落实第二期学前教育三年行动计划，启动新一轮农村义务教育学校布局调整，推进全面改善义务教育薄弱学校基本办学条件和扩充城镇义务教育资源五年计划，加强寄宿制学校建设。实施好乡村教师支持计划。加快高等教育和职业教育发展，重点支持特色骨干高校和优势特色学科建设，推动高等院校向应用型转变。规范和促进民办教育发展。办好特殊教育。完善家庭经济困难学生助学资助体系，免除建档立卡困难家庭的普通高中学生学杂费，努力让更多孩子接受更好教育!建设健康信阳。积极推进医疗与养老、保健、休闲相结合的健康产业发展。深化县级公立医院综合改革，大力支持社会办医，强化基层卫生服务能力建设。适当提高大病保险人均筹资水平和支付比例。坚持计划生育基本国策，落实全面两孩政策。提升公共服务。不断完善公共服务体系，推进基本公共服务均等化。持续开展全民健身运动，加快环浉河全民健身长廊和鸡公山体育旅游示范区建设。推进全民阅读，繁荣文艺创作，建设书香信阳。加大信阳历史文化研究，加强文化遗产保护利用。创新社会治理。着眼“双安”“双治”“双基”，加快推进法治信阳、平安信阳建设。牢固树立安全生产红线意识和底线思维，严格落实安全生产责任制和管理制度，持续加强基层基础能力建设，深化重点行业、领域专项整治，推进隐患排查和打非治违常态化，加强食品药品安全监管，坚决遏制重特大事故发生。做好信访稳定工作，依法打击处置非法集资，妥善解决群众合理诉求，防范化解各类不稳定因素。加强社会治安综合治理，健全社会治安立体防控体系，严厉打击违法犯罪，确保社会大局和谐稳定。

　　继续加强新形势下国防教育和国防后备力量建设，支持驻信解放军、武警部队、预备役部队、民兵和人民防空建设，深入开展“双拥”工作，完善涉军维权“信阳模式”，促进军民深度融合发展，巩固军政军民团结。大力支持人民团体发挥桥梁纽带作用，认真做好民族、宗教工作，切实加强外事、侨务、对台工作，继续抓好机关事务、地质、气象、防震减灾等各项工作。

　　各位代表!今年，在完成省 “十项重点民生工程”的同时，继续抓好“十件实事”，具体安排意见已经印发，请予审议。

　　四、切实加强政府自身建设

　　做好政府工作，必须落实法治政府建设实施纲要，统筹推进创新、法治、效能、廉洁型政府建设，提升政府治理能力和水平。

　　(一)加强创新政府建设。深化“放、管、服”改革，继续精简行政审批事项，清理规范行政审批中介服务事项，全面落实权责清单制度并动态管理，推开“两随机、一公开”检查，加强事中事后监管，切实把该管的真正管好、该放的彻底放开、该减的彻底减掉、该清的彻底清除;推行行政审批标准化，推广“互联网+政务服务”，深化政务公开，进一步提高行政服务质量和效率。

　　(二)加强法治政府建设。健全依法决策机制，完善公众参与、专家论证、风险评估、合法性审查等重大决策程序。推进服务型行政执法，全面落实行政执法责任制，加强行政执法监督，促进严格规范公正文明执法。启动“七五”普法。严格执行人大及其常委会决定，自觉接受人大及其常委会法律监督和工作监督，主动接受政协民主监督，认真办理人大代表建议和政协提案;广泛听取民主党派、工商联、无党派人士、人民团体意见。大力支持司法监督、社会监督和舆论监督，让人民更好地有效监督政府!

　　(三)加强效能政府建设。自觉践行“三严三实”，深入开展“两学一做”学习教育，严格落实中央八项规定精神，严格控制和管理“三公”经费，持续加强作风建设，坚决防止“四风”反弹。健全岗位履职责任制和激励机制，督促引导公务人员履职尽责、敢于担当、勤政有为、真抓实干。大力开展懒政怠政为官不为问责年活动，坚决打通政策落实中的“中梗阻”和“最先最后一公里”，努力让企业和群众办事更便捷!

　　(四)加强廉洁政府建设。深入推进惩治和预防腐败体系建设，认真履行主体责任和“一岗双责”，全面落实党风廉政建设责任制。加强廉政教育和制度建设，严守党的政治纪律和政治规矩，严格执行廉洁自律准则和各项规定，坚决把纪律挺在前面。发挥审计保障和监督作用，强化行政监察和巡视巡察监督，认真治理损害党和人民群众利益的突出问题，坚持无禁区、全覆盖、零容忍，以刮骨疗毒、壮士断腕的决心惩治腐败，以反腐倡廉的实效保障发展、取信于民!

　　各位代表!“十三五”规划纲要已为我们描绘了未来五年的蓝图，踏上新征程，加快新发展，打赢攻坚战，是时代赋予我们的光荣使命和神圣职责。让我们紧密团结在以习近平同志为总书记的党中央周围，在省委、省政府和市委的坚强领导下，以时不我待、只争朝夕的担当，发扬克难攻坚、滚石上山的精神，持续求进，务实发展，积极作为，努力实现“十三五”良好开局，为全面建成小康社会奠定更加坚实的基础!
